# Vaccination with SARS-CoV-2 inactivated vaccines reduced the risk of anxiety and depression in a population majored by health care workers during the recent omicron variant outbreak

**DOI:** 10.3389/fpsyg.2022.989952

**Published:** 2022-11-18

**Authors:** Hong Zhao, Xia Yu, Wenyi Ye, Runzhu Wang, Jifang Sheng, Yu Shi

**Affiliations:** ^1^State Key Laboratory for Diagnosis and Treatment of Infectious Diseases, National Clinical Research Center for Infectious Diseases, National Medical Center for Infectious Diseases, Collaborative Innovation Center for Diagnosis and Treatment of Infectious Diseases, The First Affiliated Hospital, College of Medicine, Zhejiang University, Hangzhou, China; ^2^Department of Traditional Chinese Internal Medicine, The First Affiliated Hospital of Zhejiang Chinese Medical University, Hangzhou, China

**Keywords:** COVID-19, omicron virus variant strain, vaccine, anxiety, depression, insomnia

## Abstract

**Background:**

The mental health status of the population majored by health care workers in China during the omicron variant outbreak remains unknown. Furthermore, the effect of COVID-19**-**inactivated vaccines on mental health is yet to be investigated.

**Methods:**

A cross-sectional, online survey study was conducted from 12–20 April, 2022. The prevalence of symptoms of depression and anxiety were evaluated using the Hospital Anxiety and Depression Scale.

**Results:**

Responses from a total of 1,387 participants were analyzed, 39.7% of which reported symptoms of mental health illness. The incidence of anxiety (30.4% vs. 48.4%, *p* < 0.001) and depression (27.1% vs. 46.3%, *p* < 0.001) decreased with COVID-19 inactivated vaccination. From multivariate analysis, living in Shanghai (anxiety: Odds ratio [OR]: 1.58, 95% confidence interval [CI]: 1.14–2.19, *p* = 0.006; depression: OR: 1.61, 95% CI: 1.16–2.25, *p* = 0.005), with a mental illness (anxiety: OR: 8.97, 95% CI: 1.01–79.56, *p* = 0.049; depression: OR: 9.32, 95% CI: 1.06–82.30, *p* = 0.045) increased the incidence of anxiety and depression. Elderly participants (anxiety: OR: 0.986, 95% CI: 0.975–0.997, *p* = 0.012; depression: OR: 0.976, 95% CI: 0.965–0.987, *p* < 0.001) who had been vaccinated against COVID-19 (anxiety: OR: 0.49, 95% CI: 0.32–0.75, *p* = 0.001; depression: OR: 0.45, 95% CI: 0.29–0.69, *p* < 0.001) had decreased incidences of anxiety and depression.

**Conclusion:**

Our findings increase the awareness of the high incidence of mental health illness symptoms during the omicron variant outbreak despite previous experiences with the COVID-19 pandemic, and vaccination is suggested to reduce the risk of anxiety and depression.

## Introduction

The pandemic of severe acute respiratory syndrome coronavirus - 2 (SARS-CoV-2) Omicron variant is a major burden of public health worldwide (Lazarus et al., 2021; Ledford, 2021; Cheung et al., 2022; Del Rio et al., 2022; Wong et al., 2022; Zhao et al., 2022). Although the initial outbreak of the coronavirus disease 2019 (COVID-19) in Wuhan was contained in 2020 in China, the highly transmissible omicron variant imposed a new threat to China (Cheung et al., 2022; Del Rio et al., 2022). Indeed, between 31 December 2021 and 25 May 2022, the fifth wave of COVID-19 in Hong Kong claimed 9,249 lives at a mortality rate of 1.25% (Cheung et al., 2022). Recently, in Shanghai cases of omicron variant infections and deaths from late February to 4 May 2022 were 60,1942 and 503, respectively, (Zhang et al., 2022).

The pandemic of severe acute respiratory syndrome coronavirus - 2 (SARS-CoV-2) Omicron variant had a severe mental health impact on the general population (Bjelland et al., 2002; Alzahrani et al., 2022; Temsah et al., 2022). According to a WHO report, the COVID-19 pandemic triggered a 25% increase in the prevalence of anxiety and depression worldwide (Kola et al., 2022). The adverse mental pressure was prominent in healthcare workers (Temsah et al., 2022). The impact of COVID-19 vaccines on individual mental health was controversial. One study reported that depression and anxiety of adults in the United States were not significantly different between periods with or without widespread availability of vaccines [The association of COVID-19 vaccine availability with mental health among adults in the United States]. However, a nationwide survey conducted in Bangladesh showed that unvaccinated populations had an increased risk of depression, anxiety, and stress symptoms compared to vaccinated populations [Psychological effects and associated factors among vaccinated and unvaccinated general population against COVID-19 infection in Bangladesh]. Further, in addition to mRNA vaccines, inactivated whole virus vaccine has been widely used to provide protection against severe COVID-19 or COVID-19-related death, especially in China. However, the effect of inactivated vaccines on mental disorders due to the pandemic of the omicron variant has been less investigated. Thus, we conducted a cross-sectional online survey study to investigate the prevalence of symptoms of depression, and anxiety in a population majored by healthcare workers during the recent omicron variant outbreak in China, and analyzed the influence of the number of vaccine doses received on mental health status.

## Materials and methods

### Study design

A large-sample, cross-sectional, online survey study involving 10 province-level regions in China was conducted from 12 April to 20 April, 2022. Shanghai was most severely affected by the Omicron variant outbreak compared with regions outside Shanghai. Thereby, we hypothesized that individuals living in Shanghai are more likely to suffer from mental disorders than those outside Shanghai. To compare the interregional differences in mental health outcomes among general people in China vaccinated with SARS-CoV-2 Vaccines during the recent Omicron variant outbreak, samples were stratified by their geographic location (i.e., Shanghai, and regions outside Shanghai). A generalized population was randomly sampled from each selected province. The target sample size of participants was determined using the formula *n* = *Z*_1-α/2_^2^ *P* (1 − *P*)/*d*^2^, in which α = 0.05 and *Z*_1-α/2_ = 1.96, and the estimated acceptable margin of error for proportion d was 0.1. The proportion of the populace with psychological comorbidities was estimated at 32%, based on a previous study of the COVID-19 outbreak (de Sousa et al., 2021). To allow for subgroup analyses, we amplified the sample size by 50% with a goal of at least 1,224 completed questionnaires from participants.

The inclusion criteria were adults of more than 18 years old living in China. The exclusion criteria were: (1) lack of information on vaccination status; (2) without informed consent. For individuals with positive SARS-Cov-2 RNA testing, genomic sequencing of viral isolates was performed to confirm the omicron variant. Data analysis was performed from April to May 2022. The study was approved by the Ethics Committee of the First Affiliated Hospital, School of Medicine, Zhejiang University, and electronic informed consent was collected before the respondents began the questionnaire. This study followed the guidelines of the American Association for Public Opinion Research Reporting.

### Data collection

A self-designed online survey was released *via* a professional survey platform ‘Wenjuanxing’. The survey link was posted on the website. It was an anonymous survey to ensure data confidentiality. A total of 1,572 individuals clicked on the survey link, and 1,434 individuals undertook the survey. Of these, 16 did not provide informed consent, four individuals came from outside of China, and 1,414 participants provided informed consent and subsequently submitted the questionnaires. Sixteen questionnaires that lacked vaccination status information were excluded from the analysis. Eleven respondents were excluded because their survey lasted more than 45 min thus their answers possibly did not accurately reflect their psychological state.

### Measurements and covariates

The survey was carried out in three parts and lasted approximately from 2 to 4 min. In the first part demographic information of the participants, including gender, age, living area, level of education, marital status, geographic region, history of chronic diseases, and occupation was collected. The second part asked pandemic-related questions, including vaccination status, number of vaccinations, and details on any prior pandemic-related isolations. The third part of the questionnaire had standardized scales. Hospital Anxiety and Depression scales (HADS) were used to assess anxiety disorders and depression in the investigation (Bjelland et al., 2002). Previous studies have demonstrated that HADS was effective in assessing mental status in Chinese populations during the COVID-19 pandemic (Yue et al., 2020; Wu et al., 2021; Alzahrani et al., 2022). The total scores of HADS were interpreted as follows: normal (0–7), suspected depression or anxiety (Bjelland et al., 2002; Alzahrani et al., 2022; Temsah et al., 2022), and depression or anxiety (Lai et al., 2020; Shi et al., 2020; Yue et al., 2020; de Sousa et al., 2021; Madison et al., 2021; Mbaeyi et al., 2021; Perez-Arce et al., 2021; Wu et al., 2021; Kola et al., 2022).

### Statistical analysis

SPSS statistical software (version 26.0; SPSS, Inc., Chicago, IL, United States) was used to analyze the data. Variables were calculated using mean ± standard deviation (SD), the median with interquartile values, numbers, or percentages. Fisher’s exact and *χ*^2^ tests were used to compare categorical variables, and Kruskal–Wallis or Mann–Whitney *U* tests were used to compare quantitative variables. Unadjusted logistic regression and multiple logistic regression analyses were used to explore factors potentially associated with depression, and anxiety. For multivariate analysis, the entry and removal probability for stepwise analysis was set at 0.05 and 0.10 respectively, and variables with *p* < 0.05 were maintained in the final model. The Forest map method for regression analysis was performed using GraphPad Prism for Windows (version 8.4.0, Graphpad Software, San Diego, California United States). In all analyses, an alpha level of 0.05 was used.

### Results

A total of 1,387 respondents were included in this study and the average age was 39.56 ± 11.48 years; most respondents (95.7%) were aged between 20 and 60. More than half (62.4%) of the respondents were healthcare workers. Among the participants, 39.7% (550/1387) reported symptoms of mental health illness. These were anxiety (31.7%) and depression (28.4%) (Table 1).

**Table 1:** Baseline characteristics of the enrolled participants living in Shanghai and outside Shanghai.

Variables	Total (*n* = 1,387)	Living in Shanghai group (*n* = 181)	Living outside Shanghai group (*n* = 1,206)	*χ*^2^/*t*	Value of *p*
**Gender (male%)**	500 (36.0%)	50 (27.6%)	450 (37.3%)	6.41	0.011
**Age (years)**	39.56 ± 11.48	38.71 ± 11.75	39.68 ± 11.44	−1.07	0.286
18–20	11 (0.8%)	2 (1.1%)	9 (0.7%)	3.58	0.310
20–40	791 (57.0%)	114 (63.0%)	677 (39.6%)		
40–60	535 (38.7%)	59 (32.6%)	476 (39.6%)		
≥ 60	49 (3.5%)	6 (3.3%)	43 (3.6%)		
**Marital status**				3.14	0.208
Unmarried	293(21.1%)	46 (25.4%)	247 (20.5%)		
Marry	1,053(75.9%)	128 (70.7%)	925 (76.7%)		
Divorced or widowed	41 (3.0%)	7 (3.9%)	34 (2.8%)		
**Educational level**				7.67	0.053
High school and below	179 (12.9%)	27 (14.9%)	152 (12.6%)		
University	889 (64.1%)	107 (59.1%)	782 (64.8%)		
Graduate and above	319 (23.0%)	47 (26.0%)	272 (22.6%)		
**Occupation**				85.05	< 0.001
Health care workers	866 (62.4%)	60 (33.1%)	806 (66.8%)		
Businessman and service	164 (11.8%)	46 (25.4%)	118 (9.8%)		
Worker	63 (4.5%)	8 (4.4%)	55 (4.6%)		
Farmer	34 (2.5%)	4 (2.2%)	30 (2.5%)		
Administrative and civilian staff	95 (6.8%)	23 (12.7%)	72 (6.0%)		
Educators	60 (4.3%)	11 (6.1%)	49 (4.1%)		
Others	105 (7.6%)	29 (16.0%)	76 (6.3%)		
**Concomitant diseases**	263 (19.0%)	29 (16.0%)	234 (19.4%)	1.17	0.279
Hypertension (*n*, %)	116(8.4%)	13 (7.2%)	103 (8.5%)	0.38	0.538
Diabetes (*n*, %)	26 (1.9%)	2 (1.1%)	24 (2.0%)	0.28	0.600
Chronic kidney disease (*n*, %)	9 (0.6%)	0	9 (0.7%)	0.45	0.503
Chronic hepatopathy (*n*, %)	27 (1.9%)	2 (1.1%)	25 (2.1%)	0.35	0.555
Heart disease (*n*, %)	19 (1.4%)	2 (1.1%)	17 (1.4%)	0.12	0.735
Immune system diseases (*n*, %)	25 (1.8%)	3 (1.7%)	22 (1.8%)	0.03	0.874
Mental illnesses (*n*, %)	6 (0.4%)	2 (1.1%)	4 (0.3%)	1.63	0.201
Tumors (*n*, %)	20 (1.4%)	3 (1.7%)	17 (1.4%)	0.07	0.789
Others (*n*, %)	70 (5.0%)	8 (4.4%)	62 (5.1%)	0.17	0.679
**Whether or not in isolation**					
Living outside of isolation	1,223(88.2%)	110 (60.8%)	1,113 (92.3%)	149.92	< 0.001
Living in location	164(11.8%)	71 (39.2%)	93 (7.7%)		
Location of isolation				295.50	< 0.001
Self-isolation at home	77 (5.3%)	55 (30.4%)	18 (1.5%)		
Isolation at hotel	61 (4.4%)	6 (3.3%)	55 (4.6%)		
Isolation at FangCang Hospital or other hospital	30 (2.2%)	10 (5.5%)	20 (1.7%)		
**Number of people in isolation with**				218.33	< 0.001
One	60 (4.3%)	15 (8.3%)	45(3.7%)		
2 to 4	54 (3.9%)	40 (22.1%)	14 (1.2%)		
More than 5	50 (3.6%)	16 (8.8%)	34 (2.8%)		
**Doses of COVID-19 inactivated vaccine**				30.61	< 0.001
0 dose	77 (5.6%)	15 (8.3%)	62 (5.1%)		
1 dose	20 (1.4%)	4 (2.2%)	16 (1.3%)		
2 doses	272 (19.6%)	61 (33.7%)	211 (17.5%)		
3 doses	1,018 (73.4%)	101 (55.8%)	917 (76.0%)		
**Anxiety (*n, %*)**	439 (31.7%)	74 (40.9%)	365 (30.3%)	8.20	0.004
**Depression (*n, %*)**	394 (28.4%)	69 (38.1%)	325 (26.9%)	9.66	0.002

Data are expressed as mean ± SD, or number (percentage). Comparisons between groups were assessed using Student’s *t*-test, Mann–Whitney *U* test, or the *χ*^2^ test.

### Demographic characteristics

Because of the omicron outbreak in Shanghai, we grouped the samples into those living in Shanghai and not living in Shanghai so as to make comparisons. Of all participants, 181 (13.1%) lived in Shanghai, and the rest lived in Zhejiang Province, Jiangsu Province, Beijing, Anhui Province, and other provinces. Among all participants, 36% were men. However, the group that did not live in Shanghai had more men participants than the group that lived in Shanghai (37.3% vs. 27.6%, *p* = 0.011). Among all participants, 62.4% were healthcare workers, and the group that did not live in Shanghai had more healthcare workers than those who did (66.8% vs. 33.1%, *p* < 0.011). However, the incidence of anxiety (31.4% vs. 32.1%, *p* = 0.803) and depression (27.9% vs. 29.2%, *p* = 0.623) were not significantly different between the healthcare workers and non healthcare workers (Supplementary Table 1). Among all participants, 11.8% had self-isolated, and more of them were living in than outside Shanghai (39.2% vs. 7.7%, *p* < 0.001). Those living outside Shanghai had lower rates of anxiety (40.9% vs. 30.3%, *p* = 0.004) and depression (38.1% vs.26.9%, *p* = 0.002) than those who lived in Shanghai. However, age, marital status, educational level, and concomitant diseases did not significantly differ between the participants living in and outside Shanghai (Table 1).

### COVID-19 vaccine and mental illness symptoms

We analyzed correlations between the number of vaccine doses received and mental health illness symptoms and found that vaccination reduced the incidence of anxiety (30.3% vs. 49.5%, *p* < 0.001) and depression (27.1% vs. 46.4%, *p* < 0.001). There were no differences in the number of doses of the COVID-19 vaccine received and gender, age, marital status, possessions of an advanced degree, ailment from mental illness and concomitant diseases, and diagnosis with SARS-CoV-2 infection (Table 2). There was a statistical difference in the number of doses of the COVID-19 vaccine received between those living in and outside Shanghai, healthcare and non-healthcare workers, and those in or outside isolation. Furthermore, with the increasing number of doses of COVID-19 vaccines, the incidence of anxiety (from 0 to 3 doses of vaccine, 49.5% vs. 30.0% vs. 29.3% vs. 30.6%, *p* = 0.001) and depression (from 0 to 3 doses of vaccine, 46.4% vs. 25.0% vs. 29.3% vs.26.5%, *p* = 0.001) decreased (Table 2).

**Table 2:** The influence of the number of inactivated vaccine doses received on anxiety and depression.

Variables	Doses of COVID-19 inactivated vaccine				*X* ^2^	*p* value
	0-dose (*n* = 97)	1-dose (*n* = 20)	2-doses (*n* = 273)	3-doses (*n* = 997)		
Gender (male%)	36 (37.1%)	5 (25.0%)	94 (34.4%)	365 (36.6%)	1.55	0.670
Age (years)	38.90 ± 11.83	37.30 ± 11.73	38.16 ± 11.05	40.05 ± 11.54	7.79	0.050
Living Shanghai	15 (15.5%)	4 (20.0%)	61 (22.3%)	101 (10.1%)	26.93	< 0.001
Marital status^†^	26 (26.8%)	6 (30.0%)	65 (23.8%)	237 (23.8%)	0.81	0.847
Educational level^§^	82 (84.5%)	18 (90.0%)	235 (86.1%)	873 (87.6%)	1.14	0.768
With mental illness	1 (1.0%)	0	1 (0.4%)	4 (0.4%)	0.81	0.848
Multiple concomitant diseases	6 (5.2%)	0	8 (2.9%)	30 (3.0%)	3.69	0.297
Occupation^‡^	48 (49.5%)	2 (60.0%)	147 (53.8%)	659 (66.1%)	21.28	< 0.001
Whether or not isolation	20 (20.6%)	3 (15.0%)	44 (16.1%)	97 (9.7%)	15.07	0.002
Isolation alone	10 (10.3%)	2 (10.0%)	12 (4.4%)	36 (3.6%)	8.59	0.035
SARS-COV-2 infection	1 (1.01%)	0	4 (1.5%)	25 (2.5%)	2.84	0.417
With anxiety	48 (49.5%)	6 (30.0%)	80 (29.3%)	305 (30.6%)	15.50	< 0.001
With depression	45 (46.4%)	5 (25.0%)	80 (29.3%)	264 (26.5%)	17.47	< 0.001

^†^: Marital status means unmarried. ^§^: Educational level means advanced degree, that is, bachelor’s degree or above. ^‡^: Occupation means comparing healthcare workers to non healthcare workers. And health care workers including doctors and nurses. Data are expressed as mean ± SD, or number (percentage). Comparisons between groups were assessed using Student’s *t*-test, Mann–Whitney *U* test, or the *χ*^2^ test.

### Risk factors of mental health with anxiety and depression

**Figure 1:**
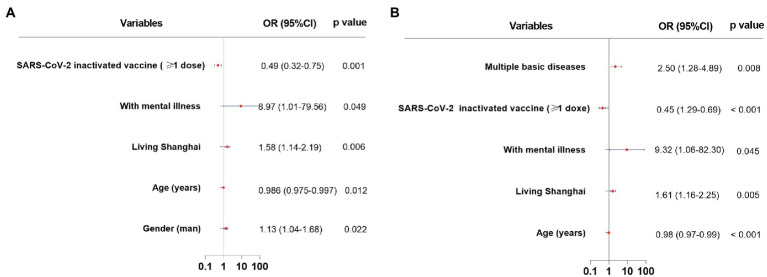
Susceptibility factors associated with anxiety and depression. **(A)**: susceptibility factors associated with anxiety. **(B)**: susceptibility factors associated with depression.

To determine susceptibility factors associated with anxiety, the following variables were used for multivariate analysis: gender, age, living in Shanghai, mental illness, multiple basic diseases, marital status, educational level, occupation, previous COVID-19 diagnosis, isolation, location of isolation, number of people isolated together with, and doses of vaccination. From multivariate analysis (Figure 1A), a man (Odds ratio [OR]: 1.33, 95% confidence interval [CI]: 1.04–1.68, *p* = 0.022), living in Shanghai (OR: 1.58, 95% CI: 1.14–2.19, *p* = 0.006), with mental illness (OR: 8.97, 95% CI: 1.01–79.56, *p* = 0.049), had increased incidences of anxiety. Elderly participants (OR: 0.986, 95% CI: 0.975–0.997, *p* = 0.012), who were vaccinated (OR: 0.49, 95% CI: 0.32–0.75, *p* = 0.001) had decreased incidences of anxiety (Supplementary Table 2).

To determine susceptibility factors associated with depression, multivariate analysis was conducted and revealed (Figure 1B) that participants living in Shanghai (OR: 1.61, 95% CI: 1.16–2.25, *p* = 0.005), with mental illness (OR: 9.32, 95% CI: 1.06–82.30, *p* = 0.045), and multiple concomitant diseases (OR: 2.50, 95% CI: 1.28–4.89, *p* = 0.008) had increased incidences of depression. Elderly participants (OR: 0.976, 95% CI: 0.965–0.987, *p* < 0.001), who were vaccinated (OR: 0.45, 95% CI: 0.29–0.69, *p* < 0.001) had decreased incidence of depression (Supplementary Table 3).

## Discussion

The present cross-sectional survey aimed to investigate the incidence of anxiety and depression in individuals during the outbreak of omicron variant infection and to assess the impact of vaccination. Our study revealed a high prevalence of anxiety (31.7%) and depression (28.0%) reported by participants. It was noted that these individuals have already experienced the COVID-19 epidemic of 2020 in China, suggesting the previous experience may not prevent episodes of mental illness impacted by the recent outbreak. Another finding was that individuals vaccinated with SARS-CoV-2 inactivated vaccines had a significantly lower incidence of mental disorders than those without, in line with the results of populations vaccinated with mRNA vaccines. This finding suggested the benefit of vaccines on mental health was irrespective of vaccine type.

Overall, 31.7, and 28.0% of all participants in the study reported symptoms of anxiety and depression, respectively. The reported incidences were consistent with a previous study in the first wave of the COVID-19 pandemic in China showing that the prevalence of symptoms of mental health illness was 31.6% for anxiety, and 27.9% for depression (Shi et al., 2020). Among participants in the study, men were more likely to develop anxiety than women. The finding was contrary to the general fact that women are more susceptible to anxiety in response to stress or psychic trauma, possibly related to unemployment or a decline in income during the COVID-19 pandemic (Lai et al., 2020; Shi et al., 2020; Temsah et al., 2022). Other risk factors of depression and anxiety included young age, a personal history of mental illnesses, and concomitant diseases associated with depression and anxiety, in accordance with prior studies (Shi et al., 2020). A notable finding was that although the incidence of anxiety and depression in our participants did not differ from that reported during the first wave of COVID-19 in 2020, these mental illness symptoms in healthcare workers were lower in our study (Lai et al., 2020). The increasing resistance to mental illness in healthcare workers may be related to their special background and increasing knowledge of COVID-19.

We also found that the residence location of individuals had an impact on the incidence of mental illness symptoms. As expected, participants living in Shanghai, the epicenter of the recent epidemic of the omicron variant in China, had a higher proportion of depression and anxiety than those who lived outside Shanghai. This finding was consistent with prior studies showing that the psychological burden of people in Wuhan in 2020 was significantly higher than that of people in other regions (Lai et al., 2020; Shi et al., 2020).

Another prominent finding was the effect the number of doses of the COVID-19 vaccine received had on mental health. Although vaccination, especially the three doses regimen, reduced the risk of critical illness and death (Lazarus et al., 2021; Mbaeyi et al., 2021; Perez-Arce et al., 2021; Cheung et al., 2022), how the number of vaccination doses received impacts a person’s mood is not clear. In this study, univariate analysis showed that vaccination reduced the incidence of anxiety and depression, although some factors including living in Shanghai, healthcare workers, living in isolation, and living alone in isolation influenced the relationship between the COVID-19 vaccine and mental health status. Using multiple logistic regression analysis, we also showed that vaccination reduced the incidence of anxiety and depression - corroborating the finding that receiving the first dose of COVID-19 vaccine significantly improved mental health (Perez-Arce et al., 2021). Psychological factors are related to the efficacy of other vaccines, leading to the hypothesis that they are related to the efficacy of COVID-19 vaccines (Madison et al., 2021). We found that COVID-19 vaccination decreased the incidence of anxiety and depression thus supporting this hypothesis.

The study had some limitations. First, our study was an online questionnaire with a cross-sectional design and lacked data on the long-term dynamic psychological changes. Second, in this study, we utilized the self-assessment scales to investigate relevant symptoms instead of standardized psychiatric diagnoses or scales administered by experienced clinicians, which may introduce bias. However, a good validity of HADS compared to other questionnaires for anxiety and depression such as BDI, STAI, CAS, and SCL-90 Anxiety and Depression subscales has been demonstrated (Bjelland et al., 2002). Third, we did not further subdivide health workers into their specific occupations thus we could not further distinguish the mental health status of personnel in different medical posts. Fourth, a skewed male:female ratio may introduce selection bias. Fifth, there is the possibility that some people, with higher anxiety and depression scores, were not vaccinated because they have hesitant, anxious, and skeptical attitudes toward vaccines, which may overestimate the anxiety and depression risk of unvaccinated people. Finally, the study was performed on adults in China and those receiving inactivated vaccines. Whether the conclusions can be extrapolated to populations outside China and those taking mRNA vaccines need further investigation.

## Conclusion

Our report demonstrated that people, mostly healthcare workers from epidemic-stricken areas are more likely to have mental health illnesses such as anxiety and depression. Even though most people experienced the impact of COVID-19 in 2020, their mental health still needs active intervention regarding facing another COVID-19 outbreak such as those caused by variants like omicron. However, vaccination with inactivated vaccines improves mental health by lowering levels of anxiety and depression.

## Data availability statement

The raw data supporting the conclusions of this article will be made available by the authors, without undue reservation.

## Ethics statement

The study was approved with the Ethics Committee of the First Affiliated Hospital, School of Medicine, Zhejiang University and written consents were collected online.

## Author contributions

HZ, YS, and JS participated in the survey study design. HZ, XY, WY, and RW collected the data. HZ, XY, and WY performed the statistical analyses. HZ and WY wrote the manuscript. YS and JS edited the manuscript. The corresponding author attests that all listed authors meet authorship criteria and that no others meeting the criteria have been omitted. HZ, XY, and WY contributed equally to this work. YS and JS are the Principal Investigators and guarantors. The corresponding authors (YS and JS) affirms that the manuscript is an honest, accurate, and transparent account of the study being reported; that no important aspects of the study have been omitted; and that any discrepancies from the study as planned have been explained. All authors contributed to the article and approved the submitted version.

## Funding

This work was supported by the National Key Research and Development Program of China (No. 2021YFC2301800) and the National Natural Science Foundation of China (Grant No. 81870425).

## Conflict of interest

The authors declare that the research was conducted in the absence of any commercial or financial relationships that could be construed as a potential conflict of interest.

## Publisher’s note

All claims expressed in this article are solely those of the authors and do not necessarily represent those of their affiliated organizations, or those of the publisher, the editors and the reviewers. Any product that may be evaluated in this article, or claim that may be made by its manufacturer, is not guaranteed or endorsed by the publisher.
