# Supplementary material for: Vaccination with SARS-CoV-2 inactivated vaccines reduced the risk of anxiety and depression in a population majored by health care workers during the recent omicron variant outbreak
Source: Front Psychol. 2022 Nov 18;13:989952. doi: 10.3389/fpsyg.2022.989952 (PMC9716876; doi:10.3389/fpsyg.2022.989952)
Supplement: Supplementary file 1 [file Data_Sheet_1.doc]

**Supplemental Tables**

**Supplemental Table 1. Baseline characteristics of the enrolled participants in health care workers and non-healthcare workers**.

| **Variables** | **Health care workers group (n=866)** | **Non-healthcare workers group (n=521)** | **χ^2^/ t** | ***p*-value** |
| --- | --- | --- | --- | --- |
| **Gender (male%)** | 270 (31.2%) | 230 (44.1%) | 23.73 | < 0.001 |
| **Age (years)** | 37.21±9.30 | 43.46±13.53 | 9.31 | < 0.001 |
| 18 - 20 | 0 | 11 (2.1%) | 116.79 | < 0.001 |
| 20 - 40 | 573 (66.2%) | 218(41.8%) |  |  |
| 40 - 60 | 284(32.8%) | 252 (48.4%) |  |  |
| ≥ 60 | 9 (1.0%) | 40 (7.7%) |  |  |
| **Marital status** |  |  | 11.72 | 0.003 |
| Unmarried | 195(22.5%) | 98 (18.8%) |  |  |
| Marry | 655 (75.6%) | 398 (76.4%) |  |  |
| Divorced or widowed | 16 (1.8%) | 25 (4.8%) |  |  |
| **Educational level** |  |  | 250.81 | < 0.001 |
| High school and below | 16 (1.8%) | 163 (31.3%) |  |  |
| University and above | 850 (98.2%) | 358 (68.7%) |  |  |
| **Concomitant diseases** |  |  |  |  |
| Hypertension (n, %) | 51 (5.9%) | 65 (12.5%) | 18.42 | < 0.001 |
| Diabetes (n, %) | 12 (1.4%) | 14 (2.7%) | 3.00 | 0.083 |
| [Chronic kidney disease](https://www.sogou.com/link?url=hedJjaC291MhqVs0KUJ_Vdj9u1Jf0w-G5oyI503zZCQ2JnfQ_VhurEg8LIhvaky7uG1s3KPZ0En8RrFgTa1SjkBc_3PGcDsH9YugdIlD8b5OXihceQ0_1J6Ctxua-XTDoB00Dh7fXVzu_CxjhvVvSA..) (n, %) | 4(0.5%) | 5 (1.0%) | 1.20 | 0.273 |
| Chronic hepatopathy (n, %) | 5 (0.6%) | 22 (4.2%) | 22.65 | < 0.001 |
| Heart disease (n, %) | 5 (0.6%) | 14 (2.7%) | 10.72 | 0.001 |
| Immune system diseases (n, %) | 15 (1.7%) | 10 (1.9%) | 0.06 | 0.800 |
| Mental illnesses (n, %) | 4 (0.5%) | 2 (0.4%) | 0.05 | 0.829 |
| Tumors (n, %) | 12 (1.4%) | 8 (1.5%) | 0.05 | 0.821 |
| Others (n, %) | 36 (4.2%) | 34 (6.5%) | 3.81 | 0.051 |
| **Living in Shanghai** | 60 (6.9%) | 121 (23.2%) | 76.13 | < 0.001 |
| **Whether or not in isolation** |  |  |  |  |
| Living outside of isolation | 101 (11.7%) | 63 (12.1%) | 0.06 | 0.810 |
| Location of isolation |  |  | 71.23 | < 0.001 |
| Self-isolation at home | 22 (2.5%) | 51 (9.8%) |  |  |
| Isolation at hotel | 55 6.4%) | 6 (1.2%) |  |  |
| Isolation at FangCang Hospital or another hospital | 24 (2.7%) | 6 (1.2%) |  |  |
| **Number of people in isolation with** |  |  | 20.85 | < 0.001 |
| One | 45 (5.2%) | 15(2.9%) |  |  |
| 2 to 4 | 20 (2.3%) | 34 (6.5%) |  |  |
| More than 5 | 36 (4.2%) | 14 (2.7%) |  |  |
| **Doses of COVID-19 inactivated vaccine** |  |  | 21.28 | < 0.001 |
| 0 dose | 48 (5.5%) | 49 (9.4%) |  |  |
| 1 dose | 12 (1.4%) | 8 (1.5%) |  |  |
| 2 doses | 147 (17.0%) | 126 (24.2%) |  |  |
| 3 doses | 659 (76.1%) | 338 (64.9%) |  |  |
| **Anxiety** (n, %) | 272(31.4%) | 167 (32.1%) | 0.06 | 0.803 |
| **Depression** (n, %) | 242(27.9%) | 152 (29.2%) | 0.24 | 0.623 |

Data are expressed as mean ± SD, or number (percentage). Comparisons between groups were assessed using Student’s t-test, Mann -Whitney U test, or the χ^2^ test.

**Supplemental Table 2** Susceptibility factors associated with anxiety

| **Variables** | **Univariate analysis** | | | **Multivariate analysis** | | |
| --- | --- | --- | --- | --- | --- | --- |
|  | **OR** | **95%CI** | **P value** | **OR** | **95%CI** | ***P* value** |
| Gender (male) | 1.34 | 1.05-1.71 | 0.020 | 1.33 | 1.04-1.68 | 0.022 |
| Age (years) | 0.99 | 0.98-1.002 | 0.090 | 0.986 | 0.975-0.997 | 0.012 |
| Living Shanghai | 1.42 | 0.96-2.09 | 0.078 | 1.58 | 1.14-2.19 | 0.006 |
| Marital status ^†^ | 0.94 | 0.69-1.28 | 0.685 | - | - | - |
| Educational level ^§^ | 0.62 | 0.42-0.91 | 0.015 | 0.70 | 0.49-1.004 | 0.053 |
| With mental illness | 8.31 | 0.93-74.14 | 0.058 | 8.97 | 1.01-79.56 | 0.049 |
| Multiple basic diseases | 1.94 | 1.00-3.75 | 0.050 | 1.89 | 0.98-3.65 | 0.057 |
| Occupation^‡^ | 1.30 | 0.97-1.74 | 0.075 | - | - | - |
| Whether or not isolation | 1.07 | 0.41-2.80 | 0.884 | - | - | - |
| Number of Isolated | 0.80 | 0.39-1.64 | 0.799 | - | - | - |
| SARS-COV-2 infection | 1.17 | 0.40-3.56 | 0.773 | - | - | - |
| Doses of inactivated vaccine |  |  |  |  |  |  |
| ≥ 1 dose | 0.65 | 0.25-1.71 | 0.384 | 0.49 | 0.32-0.75 | 0.001 |
| ≥ 2 doses | 0.64 | 0.25-1.60 | 0.337 | - | - | - |
| 3 doses | 1.14 | 0.84-1.55 | 0.388 | - | - | - |

^†^ : Marital status means unmarried.

^§^: Educational level means advanced degree, that is, bachelor's degree or above.

^‡^: Occupation means comparing healthcare workers to non healthcare workers. And health care workers including doctors and nurses.

CI: confidence interval; OR: odds ratio. Statistical analysis was performed using logistic regression analysis. For the susceptibility factors associated with anxiety, the variables entered into the multivariate analysis were gender, years, living Shanghai, mental illness, multiple basic diseases, with history or concomitant marital status, marital status, educational level, occupation, diagnosed with SARS-COV-2 infection, isolation, location of isolation, number of people isolated together, and times of vaccination.

**Supplemental Table 3** Susceptibility factors associated with depression.

| **Variables** | **Univariate analysis** | | | **Multivariate analysis** | | |
| --- | --- | --- | --- | --- | --- | --- |
|  | **OR** | **95%CI** | **P value** | **OR** | **95%CI** | ***P* value** |
| Gender (male) | 0.995 | 0.77-1.29 | 0.969 | - | - | - |
| Age (years) | 0.975 | 0.96-0.989 | < 0.001 | 0.98 | 0.97-0.99 | < 0.001 |
| Living Shanghai | 1.43 | 0.96-2.12 | 0.078 | 1.61 | 1.16-2.25 | 0.005 |
| Marital status ^†^ | 1.05 | 0.76-1.45 | 0.764 | - | - | - |
| Educational level ^§^ | 1.05 | 0.69-1.59 | 0.826 | - | - | - |
| With mental illness | 9.19 | 1.04-81.50 | 0.046 | 9.32 | 1.06-82.30 | 0.045 |
| Multiple basic diseases | 2.49 | 1.27-4.90 | 0.008 | 2.50 | 1.28-4.89 | 0.008 |
| Occupation ^‡^ | 0.95 | 0.71-1.28 | 0.752 | - | - | - |
| Whether or not isolation | 1.35 | 0.68-2.69 | 0.398 | - | - | - |
| Number of isolated | 0.72 | 0.34-1.51 | 0.379 | - | - | - |
| SARS-COV-2 infection | 0.49 | 0.14-1.68 | 0.256 | - | - | - |
| Doses of inactivated vaccine |  |  |  |  |  |  |
| ≥ 1 dose | 0.45 | 0.16-1.26 | 0.128 | 0.45 | 0.29-0.69 | < 0.001 |
| ≥ 2 doses | 1.05 | 0.39-2.84 | 0.919 | - | - | - |
| 3 doses | 0.97 | 0.71-1.32 | 0.837 | - | - | - |

^†^ : Marital status means unmarried.

^§^: Educational level means advanced degree, that is, bachelor's degree or above.

^‡^: Occupation means comparing healthcare workers to not healthcare workers. And health care workers including doctors and nurses.

CI: confidence interval; OR: odds ratio. Statistical analysis was performed using logistic regression analysis. For the susceptibility factors associated with depression., the variables entered into the multivariate analysis were gender, years, living in Shanghai, mental illness, multiple basic diseases, history or concomitant marital status, marital status, educational level, occupation, diagnosed with SARS-COV-2 infection, isolation, location of isolation, number of people isolated together, and times of vaccination.
